# Structural Analysis of Jumbo Coliphage phAPEC6

**DOI:** 10.3390/ijms21093119

**Published:** 2020-04-28

**Authors:** Jeroen Wagemans, Jessica Tsonos, Dominique Holtappels, Kiandro Fortuna, Jean-Pierre Hernalsteens, Henri De Greve, Leandro F. Estrozi, Maria Bacia-Verloop, Christine Moriscot, Jean-Paul Noben, Guy Schoehn, Rob Lavigne

**Affiliations:** 1 Department of Biosystems, KU Leuven, Kasteelpark Arenberg 21—box 2462, 3001 Leuven, Belgium; jeroen.wagemans@kuleuven.be (J.W.); jessica.tsonos@gmail.com (J.T.); dominique.holtappels@kuleuven.be (D.H.); kiandro.fortuna@kuleuven.be (K.F.); 2 Department of Biology, Vrije Universiteit Brussel, Pleinlaan 2, 1050 Brussel, Belgium; Jean-Pierre.Hernalsteens@VUB.onmicrosoft.com; 3 Structural Biology Brussels, Vrije Universiteit Brussel, Pleinlaan 2, 1050 Brussels, Belgium; Henri.De.Greve@vub.ac.be; 4 VIB Center for Structural Biology, Pleinlaan 2, 1050 Brussels, Belgium; 5 Univ. Grenoble Alpes, CEA, CNRS, IBS, F-38000 Grenoble, France; leandro.estrozi@ibs.fr (L.F.E.); maria.bacia@ibs.fr (M.B.-V.); 6 Univ. Grenoble Alpes, CNRS, CEA, EMBL, Integrated Structural Biology Grenoble (ISBG), F-38042 Grenoble, France; christine.moriscot@ibs.fr; 7 Biomedical Research Institute and Transnational University Limburg, Hasselt University, Agoralaan D, 3590 Hasselt, Belgium; jeanpaul.noben@uhasselt.be

**Keywords:** cryo-EM, jumbo phage, HK97-fold

## Abstract

The phAPEC6 genome encodes 551 predicted gene products, with the vast majority (83%) of unknown function. Of these, 62 have been identified as virion-associated proteins by mass spectrometry (ESI-MS/MS), including the major capsid protein (Gp225; present in 1620 copies), which shows a HK97 capsid protein-based fold. Cryo-electron microscopy experiments showed that the 350-kbp DNA molecule of *Escherichia coli* virus phAPEC6 is packaged in at least 15 concentric layers in the phage capsid. A capsid inner body rod is also present, measuring about 91 nm by 18 nm and oriented along the portal axis. In the phAPEC6 contractile tail, 25 hexameric stacked rings can be distinguished, built of the identified tail sheath protein (Gp277). Cryo-EM reconstruction reveals the base of the unique hairy fibers observed during an initial transmission electron microscopy (TEM) analysis. These very unusual filaments are ordered at three annular positions along the contractile sheath, as well as around the capsid, and may be involved in host interaction.

## 1. Introduction

Jumbo phages are tailed phages with a genome size of more than 200 kb [1]. Although the largest described jumbo phage, *Bacillus megaterium* phage G (498 kb), was discovered over 40 years ago, most giant phages primarily remained unnoticed, due to their large virion size in combination with classical phage isolation techniques. Generally, 0.22-µm filters are used to remove bacterial cells, thereby unintendedly also retaining bigger viral particles. Moreover, semi-solid media with relatively high agar concentration are often used. However, jumbo phages are too large to diffuse efficiently through these denser gels, so no visible plaques can be formed. In recent years though, more and more jumbo phages infecting different bacterial genera like *Bacillus, Escherichia, Klebsiella, Pseudomonas, Yersinia, Erwinia* and *Ralstonia* [2,3] have been isolated from a range of different environments, including water, soil, marine sediments, plant tissues, silkworms, composts and animal feces. Jumbo phages show diverse virion morphologies that can be much more complex than those of smaller phages [3,4,5], because they mostly contain more structural proteins, as is the case for *Pseudomonas* phages 201phi2-1 [6] and phiKZ [7]. Available capsid structures of jumbo phages usually show a canonical phage HK97 major capsid protein (MCP)-fold [8]. Additionally, large viruses can display specific structures like long, wavy and curly tail fibers, which have rarely been observed [9], and their large genomes encode DNA polymerases, RNA polymerases, endolysins, chitinases, glycoside hydrolases, lyases and many other genes with unknown functions [3]. As such, they are a distinct and diverse group of phages, but only roughly 100 different jumbo phages have been isolated since their discovery [10,11], and only a few have been structurally classified [8,12]. Therefore, the function of the proteins encoded by many uncharacterized genes they harbor, how they package such large genomes and the mechanism leading to their evolution remain largely unknown. Isolating more of these phages and studying their characteristics at the structural but also functional level could allow us to learn how such large genomes are packaged and how phages with new characteristics can be evolved or engineered, which will help us design more diverse phage cocktails against multidrug resistant bacteria. 

In this study, we used cryo-electron microscopy (cryo-EM) to determine the capsid and tail structure of jumbo coliphage phAPEC6, a phage against avian pathogenic *Escherichia coli* (APEC), causing major economic losses in poultry production worldwide. With a dimension of 136 nm, the T = 28,d head is able to carry its 350,175-bp-long viral DNA.

## 2. Results and Discussion

### 2.1. Coliphage phAPEC6 is A Jumbo Phage

**Figure 1:**
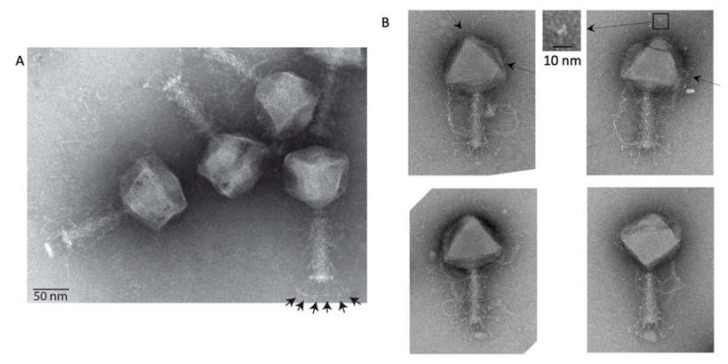
Electron microscopy of phAPEC6. (**A**) Negative staining image of phAPEC6. Six short tail fibers (arrows) on the base plate are clearly visible. (**B**) Different phAPEC6 particles display hairy appendages, sticking out from the tail but also from the capsid (arrows). Those attached to the capsid seem to be trimers (inset). The scale bar represents 50 nm.

Avian pathogenic *Escherichia coli* virus phAPEC6 was isolated during a search for candidates for phage therapy applications, but this phage drew our attention because of its large phage head, “whisker-like” tail fiber proteins and hairy appendages on the capsid and tail (Figure 1). With a diameter of 136 nm, the phAPEC6 capsid is able to carry its 350,175-bp-long viral DNA. It can thus be classified as a jumbo phage. By comparison, the coliphage 121Q head was first measured to be 116 nm [13] but corrected to be 132 nm by cryo-electron microscopy and three-dimensional reconstruction [8] and the *Klebsiella* phage RaK2 heads 123 nm for genomes of 349 and 346 kb, respectively [14,15]. 

### 2.2. Most of the phAPEC6 Genome Encodes for Unknown Proteins

**Figure 2:**
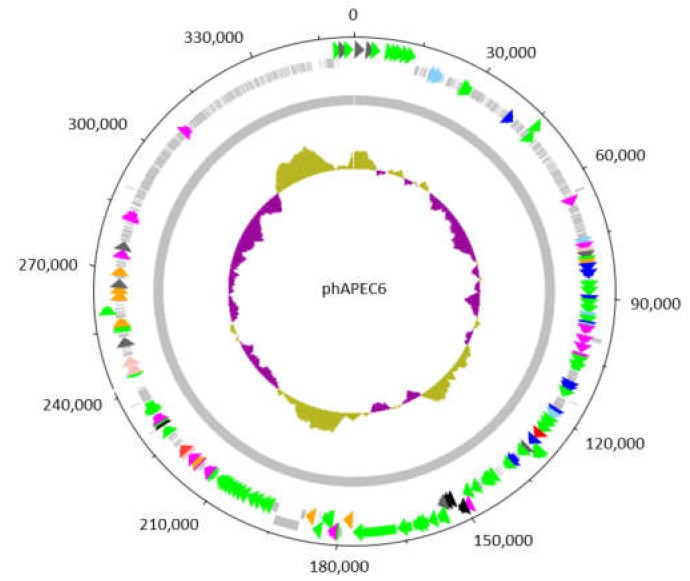
Circular representation of the genome of the giant *Escherichia* phage vB_EcoM_PhAPEC6. The inner circle represents a GC plot. The 551 identified coding sequences (CDS) are depicted by arrows or boxes if a function could be predicted or not, respectively. The 62 CDS in green, scattered along the genome, are structural proteins detected in the mature virion by mass spectrometry. The CDS indicated in light blue are exonucleases; dark blue corresponds to DNA-associated proteins such as DNA polymerases, topoisomerases, helicases and ssDNA-binding proteins; orange to proteases and dark grey to chromosome condensation domains. CDS in purple and red are involved in general metabolism and host lysis, respectively.

The phAPEC6 genome encodes 551 predicted open reading frames (Figure 2), which exceeds that of the smallest free-living bacterium, *Mycoplasma genitalium* (encoding 484 proteins) [16]. PhAPEC6 can be considered as an isolate from the same species as coliphages SP27 (97% coverage, 99.77% identity) (Azam et al., 2019; unpublished; accession LC494302.1), PBECO4 [17] (95% coverage, 98.26% identity) (accession KC295538.1) and 121Q [8,13] (96% coverage, 98.94% identity) (accession KM507819.1), belonging to the *Asteriusvirus* genus. A functional annotation of the genome (GenBank accession number MK817115) revealed that phAPEC6 encodes most DNA replication proteins, like two DNA polymerases, a clamp loader, a primase and three helicases, as well as a phage-encoded RNA polymerase sigma factor. 

Noteworthy, phAPEC6 has acquired different methods to efficiently invade its host. First, two DNA methyltransferases (N-6-adenine and C-5-cytosine) were identified, suggesting protective DNA methylation. Moreover, phAPEC6 might counter the Prr abortive infection mechanism, carried by some *E. coli* strains [18]. Large genomes often carry genes that augment or replace host functions for nucleotide synthesis, as demonstrated in the phiKZ-related giant phages [19]. PhAPEC6 is no exception on this rule, since it encodes a deoxynucleoside monophosphate kinase, a thymidylate synthase, a dCMP deaminase and a ribonucleotide reductase to make sure all nucleotides are available according to its own needs. 

However, the vast majority (83%) of predicted proteins remained unknown after in silico analysis. Therefore, mass spectrometry (ESI LC-MS/MS) analysis was performed to identify virion-associated proteins. A total of 62 different structural proteins were identified in the phage particle (Table 1)—of which, 25 have a predicted function. The specific function of the other detected structural proteins remains unknown, but most of them are unique to giant phages. Gp225 was identified as the phAPEC6 major capsid protein (MCP). This 42-kDa protein shows an identical amino acid sequence with the MCP of bacteriophage 121Q, which follow the canonical HK97 major capsid protein fold [8].

### 2.3. 3D Reconstruction of the phAPEC6 Capsid

**Figure 3:**
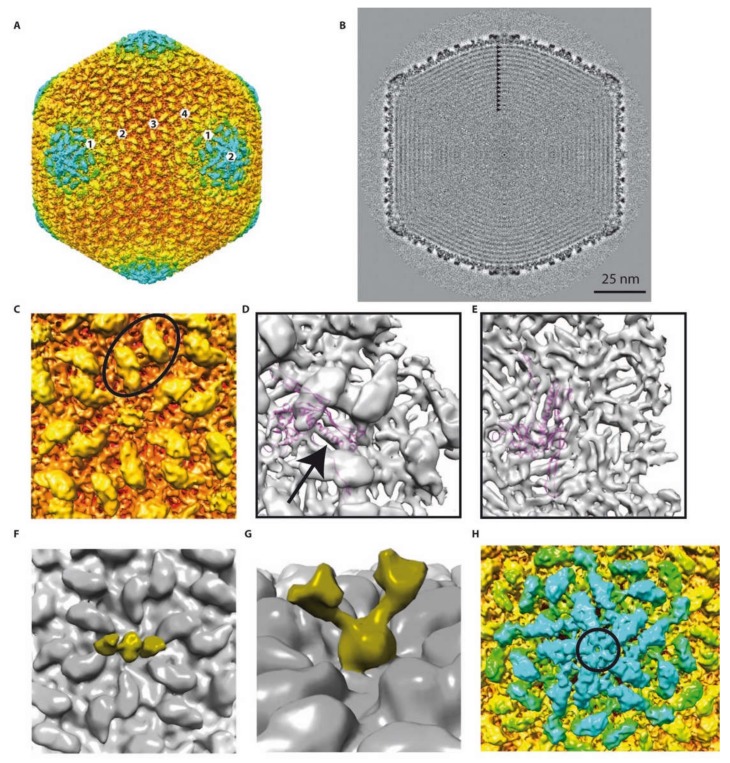
Detailed 3D reconstruction of the phAPEC6 capsid: (**A**) Isosurface representation of the phAPEC6 head 3D reconstruction at a 10-Å resolution. The particle is colored according to its diameter (from red to blue). (**B**) Central slice of the phAPEC6 head visualizing 15 concentric layers of DNA (black arrows). (**C**) Close-up view of one 6-fold axis. The major capsid protein (MCP) is colored in orange and the decoration protein in yellow. The black oval highlights a local 2-fold axis. (**D**) Fitting of the HK97 MCP X-ray structure into the phAPEC6 hexamer density seen from the outside of the particle (an arrow highlights the long alpha helix present in both HK97 and phAPEC6 MCP) and (**E**) seen from the inside of the particle. (**F**) Detail of the hexamer central protein along a 2-fold axis, displaying the fiber-anchoring protein. (**G**) Side view of the fiber-anchoring protein. (**H**) Close-up view of the 5-fold axis. The pentamer is colored in blue and highlighted by a black circle. The same protein as the one colored in yellow in part C is visible around the 5-fold axis.

The phage capsid structure was determined using cryo-EM and image analysis. The three-dimensional structure of the head has been solved to 10 Å resolution (Figure 3A). The shell is composed of several layers of protein. A 40-Å-thick nearly continuous layer is made of the MCP (Figure 3B). This shell is following icosahedral symmetry with local six-fold symmetry axes located on the facets and five-fold symmetry axes on the vertices. The capsid has a triangulation number of T = 28 (defined as T = h^2^ + hk + k^2^), since the path between two pentamers requires four steps over the h axis (h = 4) and two steps over the k axis (k = 2) going to the right (*dextro*). The determination of the chirality (*dextro* vs. *laevo*) was possible thanks to the fitting of the HK97 MCP X-ray structure into the EM density (Figure 3D,E).

Thus, the phAPEC6 phage head is constituted of 1620 major capsid proteins ((282 − 12)*6), according to Baker, Olson and Fuller [12]. The pentameric vertex proteins are probably made by another capsid protein (circle in Figure 3H) that remains unidentified given the attained resolution. The giant *Pseudomonas* phage phiKZ, as well as RSL1, on the other hand, are built of 1560 HK97-like MCP monomers, with T = 27 [20,21].

The available X-ray structure of the phage HK97 MCP has been fitted into the EM map (Figure 3D,E). The quality of the fit (see, for example, the long alpha helix; arrow in Figure 3D) suggests that the phAPEC6 MCP belongs to the HK97-fold family. One main difference is, of course, the geometry of the hexamer. Due to the difference in diameter of the two bacteriophages, the radius of curvature is much smaller in phAPEC6 compared to HK97, which make the hexamer flatter.

At the vertex level (excluding the one on which the tail is connected, i.e., Gp230), the MCP seem to be absent and replaced by a more globular protein, forming a dome-like structure. However, no putative vertex protein was found for phAPEC6 in silico. 

There is a second layer of protein stabilizing the capsid shell. This protein is elongated, forming a hexagonal network on the surface of the MCP and interacting with its neighbor over the local two-fold axis (oval in Figure 3C). This protein is also present at the vertex level and in association with the globular one closing the vertex. This protein is present in as many copies as the MCP but cannot be assigned based on mass spectrometry analysis. Even if the HK97 and phAPEC6 MCP are of the same size, it cannot be completely excluded that this cylindrical density is an additional domain of the MCP.

The last visible protein is a fibrous protein anchored in the center of each hexamer and protruding out from the shell. Even if no symmetry was imposed at the hexamer level, the extension clearly exhibits a dimeric shape: two arms are visible at a lower threshold (Figure 3F,G). At a higher contour level, the fibers are more difficult to see and are not continuous, probably because of their flexibility. However, in negative staining, fibers decorating the capsid are visible (Figure 1B top-left), and in some cases (Figure 1B top-right), the trimeric nature is recognizable (triskelion shape). No similarity with known decorating proteins was identified among the predicted proteins; hence, these fibers probably account for some of the functionally unassigned proteins of this phage. 

A triangulation number T = 28,d like the one of phAPEC6 has only been encountered in the jumbo *Escherichia coli* phage 121Q [8]. This phage has a head dimension of 132 nm, very close to that of phAPEC6. Structural comparison between phAPEC6 and the coliphage 121Q did not reveal significant differences at the current resolution: the capsid but, also, all the decorating proteins are identical. This confirms the fact that the two viruses, even if isolated and purified independently, are two isolates of the same species. 

The size of the phage head is mainly determined by the number of major capsid proteins, as the size of this protein does not vary substantially among different phages. For example, HK97 contains a genome of 40 kb in a shell with T = 7, composed of 31-kDa major capsid proteins, processed from a 42-kDa protein. A much-higher triangulation number is necessary to contain the 351-kb phAPEC6 genome in a shell built of the same-size capsid proteins (Gp225, 42 kDa). For phage phiKZ, as well as RSL1, with only a difference of 60 monomers with phAPEC6 (T = 27 vs. T = 28), the difference in diameter can be quite important (123 nm vs. 136) [20,21].

HK97 lacks a scaffolding protein that promotes inter-subunit interactions for particle formation. This function is instead fulfilled by a segment of the major capsid protein [22]. PhAPEC6, on the contrary, possesses a putative scaffolding protein, Gp226. Furthermore, a subunit of the DNA topoisomerase (Gp159) was detected as part of the structural proteome. The presence of proteins in the phage head without a structural function is not rare. The major characteristic of N4-like phages is their large RNA polymerase carried by the mature virion [23]. Moreover, a DNA topoisomerase has also been identified in the proteome of a jumbo *Ralstonia solanacearum* phage [24]. The detected single-stranded DNA binding protein (Gp204) in the phAPEC6 particle is putatively involved in origin-dependent and recombination-dependent replication, like its T4 counterpart Gp32 [25]. 

Organization of the genomic phAPEC6 DNA indicates a high density of the packaged DNA (Figure 3B). The DNA molecule is highly organized, packed in concentric layers. At least 15 clear layers were identified (arrows in Figure 3B), each separated by ~23Å. This large number is in agreement with the DNA length. In comparison, in phage T4, only eight layers have been visualized 25 Å apart from each other [26], and phiRSL1 has 13 visible layers for a DNA of size 240-kb [21]. 

**Figure 4:**
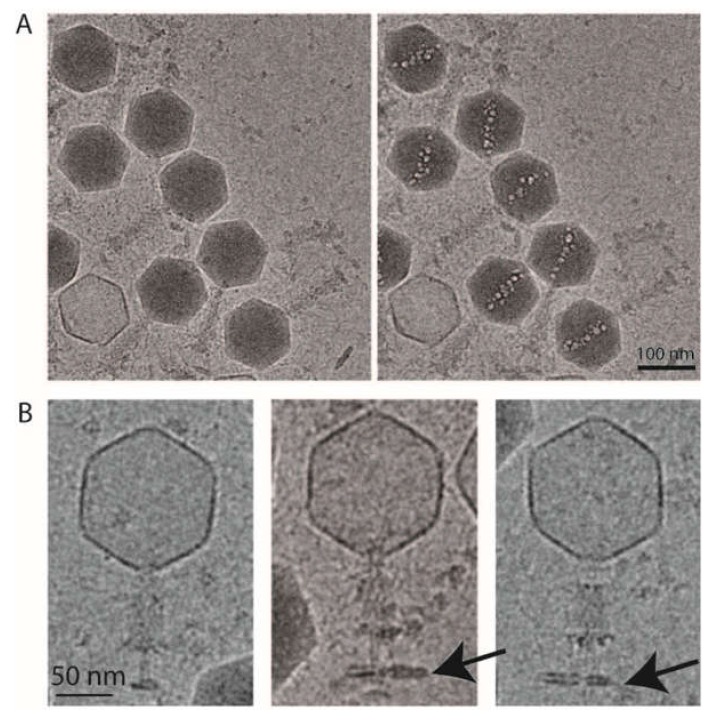
Cryo-electron microscopy of phAPEC6. (**A**) Low-dose and high-dose cryo-electron microscopy images of phAPEC6 demonstrate the presence of an inner body. (**B**) Cryo-electron microscopy images of the contracted form of phAPEC6. The capsid has released all the DNA, the tail is contracted and the presence of a little piece of host cell membrane is visible (arrow).

Under a high electron dose (300 e^-^/Å^2^), the cryo-EM images revealed the formation of bubbles in the head of phAPEC6 (Figure 4A). A cylindrical protein structure became visible, which is called the inner body. This structure, 91-nm-long and 18-nm-wide, is oriented nearly perfectly along the portal axis and becomes visible due to the formation of gaseous bubbles, resulting from the disruption of radiation-sensitive proteins. It is assumed that their sensitivity results from the fact that these proteins are embedded in DNA, as no bubbling is observed in DNA-free particles. This characteristic bubblegram has only been described in phiKZ-like phages and in coliphage 121Q [8,27,28,29], although internal protein structures (a core) have also been observed in several phages, like T7 and the *Salmonella* phage epsilon15. The genomes of those podoviruses are evenly wound around the core, which is involved in the DNA injection process [30]. 

This radiation-sensitive cylindrical structure in phiKZ is thought to be also involved in DNA ejection and packaging. Rapid and efficient ejection of such a large genome requires a highly ordered structure, which would be provided by inner-body proteins with DNA spooled around them [31]. The inner body of phiKZ is constituted of six different proteins. However, none of those phiKZ inner-body proteins shows sequence similarity to any of the phAPEC6 proteins. Among the proteins with predicted functions, the ones with a “regulator of the chromosome condensation (RCC1)” domain, might have the highest potential in the creation of an ordered DNA structure because of their known interaction with DNA. RCC1 proteins have been identified in the genomes of many eukaryotes, forming interactions with nucleosomes and regulating the condensation of DNA [32]. Unfortunately, no information is available on their function in prokaryotic organisms. Only one phage-encoded RCC1-like protein was found in the related GAP32 (GAP32 Gp1). Surprisingly, phAPEC6 encodes eleven similar RCC1-like proteins, suggesting an abundant role in phage biology.

### 2.4. 3D Reconstruction of the Entire Phage Tail

**Figure 5:**
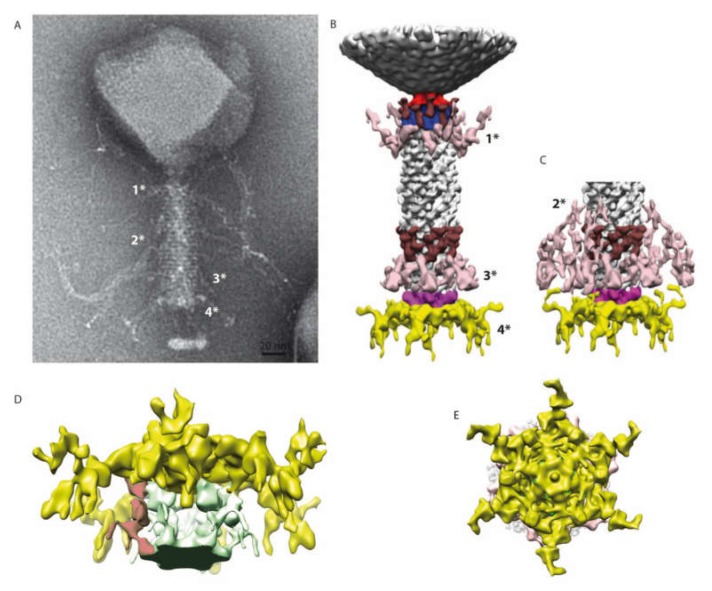
3D reconstruction of the entire tail. (**A**) A negative staining image of phAPEC6. The different fibers are labeled 1*, 2*, 3* and 4* (for the baseplate one). (**B**) 3D reconstruction of the entire tail at a normal threshold (resolution 25 Å). The baseplate plus fiber are colored in yellow and pink; the different fibers anchored in the tail (1*, 2* and 3*) are in light pink; a decoration protein present at 2 levels of the tail is colored in brown and the collar and the portal are colored respectively in blue and red. (**C**) Distal part of the tail at a high contour level: the fibers located at the 2*-level are visible. The same color code as in B has been used. (**D**) Detail of the baseplate at a higher contour level. The host membrane patch (green), as well as some fibers connected to the membrane, are visible (red). (**E**) Bottom view of the baseplate showing the complex organization of this part.

The structure of the tail of phAPEC6 was also determined by single-particle image analysis imposing a C6 symmetry. The 3D structure determined only at around a 25-Å resolution can be divided in three different parts: the collar, the contractile tail and the baseplate (Figure 5). The capsid is connected to the tail via a classical collar made of a double-hexameric ring (Figure 5B, blue part) and a connector (red in Figure 5B). Based on our MS analysis, Gp280 has sequence similarity with a neck protein and is thus part of the neck section of phAPEC6.

The collar is decorated by 12 elongated proteins (brown). Some fibers are also anchored just below the collar (pink 1*). The same pair of structures (12 brown elongated proteins and, just underneath, the same kind of pink fibers 3*) can also be found on the opposite part of the tail. The contractile tail section (94-nm-long) is built by 25 hexameric stacked rings of the identified tail sheath protein (Gp277). Upon receptor recognition, contraction of those tail sheath proteins results in phage DNA injection into the host. They represent, together with the major capsid protein, the most abundant structural proteins. The predicted molecular weight of the phAPEC6 tail sheath monomers is 97 kDa. 

The three-dimensional reconstruction allows determining the base of the hairy fibers observed during TEM analysis. These very unusual filaments are ordered in rings at three positions along the contractile sheath (light pink in Figure 5B,C; they are called 1*, 2* and 3*). Two fiber locations are visible at a normal isosurface threshold, but the third one only becomes visible at a higher contour level (Figure 5B, right). This, together with the fact that only the start of the fibers is visible, indicates that they are not rigid enough to allow their complete visualization after image analysis averaging. The length and fibrous nature of these “hairy” proteins support the hypothesis of their role during the host interaction, possibly by entanglement. Decoration proteins of the same shape and size as the one present at the collar level can also be found at the distal part of the tail.

At the baseplate, six tail fibers can be observed (Figure 1A, Figure 3B–E, Figure 4). In silico, different putative tail fiber proteins have been identified: Gp248, Gp256 and Gp314. All three proteins were recovered in the MS data. From the two predicted lysozymes, Gp241 and Gp293, only Gp241 could be detected by MS. Finally, the putative baseplate wedge protein Gp44 was identified in the structural proteome. The putative tail fiber assembly protein, Gp220, could not be detected by LC-MS/MS. In the isosurface view of the tail part, one can notice the presence of a lipid patch containing probably the bacterial receptor (arrow in Figure 4B). This kind of structure has already been observed for other phages like P2 [33]. In the 3D reconstruction at a high contour level (Figure 5D), visualization of this small membrane piece is possible. Moreover, it is also possible to observe the fibers connected to the membrane and, therefore, shed light onto the first step of infection (red part in Figure 5D).

In conclusion, apart from the extreme large phage G, phAPEC6 is, together with 121Q, an isometric bacterial virus with one of highest triangulation numbers (T = 28,d). Hua et al. [8] postulated that one way the jumbo phage genomes have expanded is through tandem duplication of preexisting genes. The presence of an array of closely related genes in phAPEC6 appears to confirm this hypothesis. Although we have established the structural basis for the tail-associated “hairy” whiskers, their specific role remains to be elucidated. However, the recent identification of the *Erwinia*, *Pectobacterium* and *Cronobacter* infecting phage CBB suggests a role in more widespread ecological niches [34]. From an applied perspective, this phage has been shown to encode several enzymes associated with biofilm and exopolysaccharide degradation and cell lysis, in addition to replication and transcription enzymes, all of which can be exploited as antimicrobial enzymes and as biotechnological tools [35,36]. 

## 3. Materials and Methods 

### 3.1. Molecular Analysis of phAPEC6

Bacterial virus PhAPEC6 (vB_EcoM_PhAPEC6) was isolated serendipitously as an impurity associated with an N4-related phage, isolated from a farm environment within the context of establishing phage cocktails targeting APEC infections [37]. PhAPEC6 was purified using CsCl gradient ultracentrifugation, as described previously [37]. Subsequently, genomic DNA was isolated and sequenced using the Illumina MiSeq platform. This generated two-times 2,844,230 reads and a 53.3 x coverage after trimming and assembly using Shovill [38]. The phage genome was annotated according to Adriaenssens et al. [39]. The large terminase shows the highest homology to the corresponding proteins from coliphages T4 and RB49 and *Vibrio* phage KVP40. Therefore, phAPEC6 has a headful packaging strategy, according to Merrill et al. [40]. Since there are no real physical ends for these types of genomes, the starting point of the genome was chosen by alignment to *Cronobacter* phage GAP32 [41], the closest characterized phage at the time of analyzing the phAPEC6 genome.

To analyze the structural proteome, 1-mL methanol and 750-µl chloroform were added to 1-mL 10^11^ purified phages. After 5 min centrifugation at 16,000× *g*, the top layer was removed, and the same amount of methanol was added once more. The sample was centrifuged again; after which, the supernatant was removed. The pellet was air-dried, resuspended in SDS-PAGE-loading buffer and loaded on a 12% polyacrylamide gel. After migration, the gel was Coomassie-stained. Across the complete length of the lane, gel slices were picked and trypsinized [42] for further mass spectrometry analysis using LC-MS/MS on an Easy-nLC 1000 liquid chromatograph (Thermo Scientific, Waltham, MA, USA) that was online-coupled to a mass-calibrated LTQ-Orbitrap Velos Pro (Thermo Scientific), as described previously [43]. The analysis of the mass spectrometric RAW data was carried out using Proteome Discoverer software v.1.3 (Thermo Scientific).

### 3.2. Electron Microscopy

For TEM, the phages were stained using 2% ammonium molybdate, pH 7.5, and imaged in a T12 FEI EM using an Orius SC1000 CCD camera according to the method described by Mas et al. [44]. 

For cryo-EM, on the other hand, the CsCl in the sample was removed using 100-kDa cutoff centricon filter units (Amicon, Merck Millipore, Burlington, MA, USA). Four microliters of sample were loaded onto a glow-discharged Quantifoil R2/1 holey grid (Quantifoil Micro Tools GmbH, Großlöbichau, Germany), vitrified using a Mark IV vitrobot (FEI) (blot force 1, 2s, 100% humidity and 20 °C). The frozen grid was transferred onto a Polara electron microscope working at 300 kV. The images were taken manually under low-dose conditions (less than 20 e-/Å2) and with a nominal magnification of 31,000 on KODAK SO-163 films. The negatives were developed in a full-strength D19 developer for 12 min [42]. Selected micrographs have been digitized on a Zeiss scanner (photoscan TD) at a step size of 7 µm, giving a pixel size of 2.25 Å.

### 3.3. Phage Head Reconstruction

Phage head images have been analyzed with the model-based PFT2/ EM3DR2 package [45]. Particles (6915) have been selected by hand into 587 × 587 pixels^2^ boxes using X3D [46] and corrected for the Contrast Transfer Function (CTF), as described previously (CTFMIX, 44). The 3D structure of phiRSL1 [21] has been scaled to the same size as phAPEC6, low-pass-filtered to 45 Å and used as the starting model. Determination of particle origin and orientation were performed with the model-based PFT2 programs using an RSL1 [21] low-resolution structure as the starting model. The finer angular step used for the refinement was 0.2 degrees. Particles (4149) out of 6915 were used for the final reconstruction and the final resolution. The resolution of the final map was estimated to be around 10 Å by the Fourier shell correlation [47] calculated between independent half-dataset maps and applying a correlation limit of 0.5 (not shown). Map visualization and HK97 MCP fittings were done with CHIMERA [48].

### 3.4. 3D Reconstruction of phAPEC6 Full Tail

Due to overlapping with other phage particles, bad orientations and partial views, only 1024 tails images were selected into 988 × 988 pixel^2^ boxes. These full tail images were phase-flipped according to the CTF, binned two times and subjected to projection-matching image analysis imposing only 6-fold symmetry using SPIDER [49]. Five-hundred images out of 1024 were including in the 25-Å resolution final reconstruction.

## Figures and Tables

**Table 1:** Structural proteome of PhAPEC6.

Protein	Function	MW (kDa)	Unique Peptide Count	Sequence Coverage (%)
gp003	DNA condensation protein	41.39	4	11.6
gp005	DNA condensation protein	40.25	2	5.7
gp006	hypothetical protein	41.36	1	3.3
gp007	DNA condensation protein	30.06	3	17.5
gp009	DNA condensation protein	39.30	3	10.8
gp010	hypothetical protein	41.39	1	3.1
gp035c	chromosome segregation protein	46.39	8	28.8
gp037c	hypothetical protein	14.83	5	48.0
gp077	hypothetical protein	42.88	10	37.8
gp078c	hypothetical protein	29.73	4	21.4
gp149c	hypothetical protein	25.61	4	29.2
gp150c	hypothetical protein	21.33	5	34.8
gp158c	hypothetical protein	90.30	9	17.1
gp159c	topoisomerase II small subunit	54.54	2	8.1
gp161c	hypothetical protein	28.98	6	28.9
gp162c	hypothetical protein	60.05	10	25.4
gp167c	hypothetical protein	20.66	4	23.4
gp179c	hypothetical protein	21.48	2	15.0
gp182c	hypothetical protein	12.33	1	11.7
gp192c	hypothetical protein	36.90	7	32.0
gp203c	RecA-like recombination protein	41.58	2	6.7
gp204c	single-stranded DNA binding protein	38.89	4	17.1
gp205c	hypothetical protein	48.48	2	8.4
gp210	hypothetical protein	20.92	6	69.8
gp211	hemagglutinin repeat-containing protein	24.30	13	69.9
gp215c	hypothetical protein	26.56	5	26.3
gp225c	major head protein	42.18	14	74.9
gp226c	scaffolding protein	42.21	2	4.9
gp227c	prohead core scaffold and protease	23.01	2	15.0
gp228c	hypothetical protein	31.12	5	16.7
gp230c	portal vertex protein of head	64.56	9	21.1
gp231c	hypothetical protein	79.72	1	1.4
gp238	hypothetical protein	27.50	2	12.8
gp239	hypothetical protein	12.37	1	8.7
gp240	hypothetical protein	85.40	5	10.0
gp241	lysozyme family protein	95.90	7	9.9
gp244	baseplate wedge	131.18	21	24.2
gp245	hypothetical protein	381.88	17	7.6
gp248	proximal tail protein	51.67	4	13.9
gp250c	hypothetical protein	40.33	9	40.2
gp251c	hypothetical protein	21.95	2	13.4
gp253	hypothetical protein	40.99	14	65.6
gp256	tail fiber protein	180.38	15	15.9
gp259c	thymidylate synthase	35.51	3	10.7
gp262c	hypothetical protein	25.02	1	6.4
gp265c	ATPase	49.52	6	19.0
gp267c	hypothetical protein	35.96	1	4.9
gp268c	hypothetical protein	51.12	6	17.8
gp270c	hypothetical protein	16.20	4	41.1
gp273c	hypothetical protein	40.42	7	29.9
gp274c	hypothetical protein	28.76	3	16.3
gp275c	hypothetical protein	25.07	2	9.2
gp277c	tail sheath monomer	97.05	36	68.0
gp280c	neck protein	29.93	4	20.5
gp306c	hypothetical protein	16.90	6	65.8
gp314c	tail fiber protein	105.34	8	13.0
gp315c	hypothetical protein	4.66	2	69.0
gp323c	hypothetical protein	98.62	4	5.6
gp341c	PhoH-like protein	53.21	8	30.9
gp347	hypothetical protein	14.10	8	75.2
gp549	DNA condensation protein	41.77	4	13.2
gp551	DNA condensation protein	41.23	5	14.2

## Data Availability

The phAPEC6 genome annotation data is available in GenBank through accession number MK817115.Cryo-EM maps have been deposited in the Electron Microscopy Data Bank: phAPEC6 capsid map EMD-10926 and phAPEC6 tail map EMD-10929.
